# CCR7 Expressing Mesenchymal Stem Cells Potently Inhibit Graft-versus-Host Disease by Spoiling the Fourth Supplemental Billingham’s Tenet

**DOI:** 10.1371/journal.pone.0115720

**Published:** 2014-12-30

**Authors:** Hong Li, Yan-Ming Jiang, Yan-Feng Sun, Ping Li, Rui-Jie Dang, Hong-Mei Ning, Yu-Hang Li, Ying-Jie Zhang, Xiao-Xia Jiang, Xi-Min Guo, Ning Wen, Yan Han, Ning Mao, Hu Chen, Yi Zhang

**Affiliations:** 1 Department of Cell Biology, Institute of Basic Medical Sciences, Beijing 100850, People’s Republic of China; 2 Department of Hematopoietic Stem Cell Transplantation, Affiliated Hospital to Academy of Military Medical Sciences, Beijing 100071, People’s Republic of China; 3 Department of Ophthalmology, The Second Artillery General Hospital, Beijing 100088, People’s Republic of China; 4 Department of Pediatrics, General Hospital of Chinese People's Armed Police Forces, Beijing 100039, China; 5 Department of Stomatology, Chinese PLA General Hospital, Beijing 100853, China; 6 Department of Plastic and Reconstructive Surgery, Chinese PLA General Hospital, Beijing 100853, China; Penn State University, United States of America

## Abstract

The clinical acute graft-versus-host disease (GvHD)-therapy of mesenchymal stem cells (MSCs) is not as satisfactory as expected. Secondary lymphoid organs (SLOs) are the major niches serve to initiate immune responses or induce tolerance. Our previous study showed that CCR7 guide murine MSC line C3H10T1/2 migrating to SLOs. In this study, CCR7 gene was engineered into murine MSCs by lentivirus transfection system (MSCs/CCR7). The immunomodulatory mechanism of MSCs/CCR7 was further investigated. Provoked by inflammatory cytokines, MSCs/CCR7 increased the secretion of nitric oxide and calmed down the T cell immune response *in vitro*. Immunofluorescent staining results showed that transfused MSCs/CCR7 can migrate to and relocate at the appropriate T cell-rich zones within SLOs *in vivo*. MSCs/CCR7 displayed enhanced effect in prolonging the survival and alleviating the clinical scores of the GvHD mice than normal MSCs. Owing to the critical relocation sites, MSCs/CCR7 co-infusion potently made the T cells in SLOs more naïve like, thus control T cells trafficking from SLOs to the target organs. Through spoiling the fourth supplemental Billingham’s tenet, MSCs/CCR7 potently inhibited the development of GvHD. The study here provides a novel therapeutic strategy of MSCs/CCR7 infusion at a low dosage to give potent immunomodulatory effect for clinical immune disease therapy.

## Introduction

Graft-versus-host disease (GvHD), a representative of T cell-mediated immune responses, remains a significant cause of morbidity and mortality in patients undergoing bone marrow transplantation. Billingham’s tenets reflect the three basic principles in the development of GvHD [1]. Additionally, some investigations highlighted that the effector cells migrating to the target tissues is important for the development of GvHD. FTY720 inhibited GvHD lethality by preventing lymphocyte egress from Secondary lymphoid organs (SLOs) to peripheral organs [2]–[4]. Corticosteroids, the first-line therapy of GvHD, make lymphocytes trafficking into bone marrow, but away from lymph nodes and inflammatory organs. All these proved the critical role of the lymphocyte homing requirement in the GvHD development, which was proposed as a corollary to Billingham’s criteria [5]. Meanwhile, this provided chances to modulate GvHD by controlling lymphocyte trafficking [6], [7].

Mesenchymal stromal cells (MSCs) are multipotent non-hematopoietic progenitor cells of stromal origin that can be isolated from the bone marrow or other tissues (adipose tissue, cord blood) [8]–[11]. MSCs have potent immunomodulatory effects. When cultivated with dendritic cells (DCs), T-lymphocytes and NK cells, they can shift them to the anti-inflammatory phenotypes. Some soluble factors participate in this processes, such as IL10, nitric oxide (NO), indoleamine 2,3-dioxygenase, prostaglandin E2, etc [12]–[14]. Therefore, MSCs have been employed to treat various immune disorders in animal models and clinical settings.

SLOs, including spleen (SP), lymphoid nodes (LN), mesenteric lymphoid nodes (MLN), Peyer’s Patches (PP), etc, are‘hubs’of immune surveillance [15], [16]. Our previous study showed that CCR7 guide the migration of MSCs to SLOs, separate GvHD from GvL effect [17]. In this study, we further demonstrated that the inducible immunomodulatory activity *in vitro* of MSCs/CCR7 is depending on the NO production. Transfused MSCs/CCR7 relocate at the appropriate T cell-rich zones within SLOs and inhibit GvHD lethality through spoiling the fourth supplemental Billingham’s tenet.

## Materials and Methods

### Ethics statement

This study was carried out in strict accordance with the recommendations in the national guidelines for the use of animals in scientific research ‘‘Regulations for the Administration of Affairs Concerning Experimental Animals’’. The protocol was also approved by the Animal Care and Use Committee of Beijing Institute of Basic Medical Sciences (Permit Number BMS-1104139), and all efforts were made to minimize suffering.

### Mice

Inbred BALB/c (H-2d) and C57BL/6 (H-2b) male mice were purchased from the Laboratory Animal Center, Academy of Military Medical Sciences. Animals were maintained under specific pathogen-free conditions and all animal experiments were performed in accordance with the Academy of Military Medical Sciences Guide for Laboratory Animals.

### MSCs culture

Primary MSCs were isolated from murine compact bone and culture-expanded as described in our previous report [18], and grown in minimal essential medium (MEM, Gibco) with 4 mM L-glutamine, 100 ^U^/ml penicillin, 100 ^U^/ml streptomycin and 10% fetal bovine serum (FCS) in a humidified atmosphere of 5% CO_2_ at 37°C.

### Reverse transcription-polymerase chain reaction (RT-PCR)

Murine MSCs derived from compact bone at passage 4 were collected for murine CCR7 detection. Splenic cells (SPC) from the same species served as positive controls. Human MSCs derived from bone marrow (hBM-MSCs, Cyagen) or umbilical cord (hUC-MSCs, Cyagen) at passage 5 were obtained for human CCR7 expression analysis. Human peripheral blood cells (hPBC) were served as positive control. The specific PCR primers were listed as followed. Murine CCR7: 5′-CAGCCTTCCTGTGTGATTTC-3′ (forward), 5′-TGGGAGAGGTCCTTGTAGTC-3′ (Reverse); Human CCR7: 5′-CCAGACAGGGGTAGTGCGAG-3′(Forward), 5′-AGGCAGAAGAGTCGCCTATG-3′ (Reverse); Murine GAPDH: 5′-GGAGCGAGACCCCACTAACA-3′ (Forward), 5′-ACATACTCAGCACCGGCCTC-3′ (Reverse); Human GAPDH: 5′-ATGGGGAAGGTGAAGGTCGGAGTCAA-3′ (Forward), 5′-CGGAGGGGCCATCCACAGTCTTCT-3′ (Forward). RT-PCR was performed as described by the manufacture (TOYOBO).

### Lentiviral transduction

Murine MSCs were seeded in serum and antibiotic-free medium. The next day, MSCs were transduced with lentivirus (Invitrogen) expressing murine CCR7 (MSCs/CCR7-eGFP) or control lentivirus (MSCs/eGFP) in the presence of 10 µg/ml polybrene (Sigma) for 6 hours.

### Flow cytometry (FCM) analysis

Phycoerythrin (PE) conjugated monoclonal antibodies against mouse CD3 (clone 145-2C11) was purchased from BD-Pharmingen. PerCP and Alexa647 conjugated monoclonal antibodies against mouse CD62L (MEL14), CCR7 (4B12) were from BioLegend. For cell surface CCR7 detection, cell surface FcγIIIR/FcγIIR was pre-reacted with purified anti-mouse CD16/32 (clone 93). Cells were collected on a FACSCalibur with CellQuest software (BD Biosciences). Data were analyzed using Flowjo 7.6.

### Inducible nitric oxide synthase (iNOS) detection [19]

MSCs, MSCs/eGFP and MSCs/CCR7 were planted on the microscope cover glasses (NEST) in the 24-well plate overnight and treated with IFNγ plus TNFα (2 ng/ml each) for another 72 hours. Then cells were collected for immunofluorescence detection using the polyclonal iNOS antibody, followed by PE goat anti–mouse IgG (BD Transduction Laboratories). Confocal images were collected by the Zeiss LSM510 Meta and were acquired using a LSM image browser.

### Detection of NO

NO in culture supernatants was detected using a modified Griess reagent (Sigma-Aldrich). Briefly, all NO_3_ is converted into NO_2_ by nitrate reductase, and total NO_2_ detected by the Griess reaction. NaNO_2_ served as a standard.

### Carboxyfluorescein diacetate succinimidyl ester (CFSE) staining

CD3^+^T cells selected with CD3ε MicroBead Kits (MiltenyiBiotec) were labeled with 5 µM CFSE (Invitrogen) for 8 min at 37°C with gentle vortex every 2 min. The labeling was terminated by adding equal volume of FCS. After washing, cells were cultured with different dose of MSCs/eGFP or MSCs/CCR7 in the presence of 50 ng/ml phorbol 12-myristate 13-acetate (PMA, Sigma) and 1 µg/ml ionomycin (ION, Sigma) for 48 hours. Cell division, as evidenced by reduction of fluorescence intensity, was analyzed by FCM.

### *In vivo* distribution of transplanted MSCs

In order to detect the specific anatomic distribution within SLOs of transplanted MSCs/eGFP or MSCs/CCR7, cells (5×10^5^) were injected into the lateral tail vein of GvHD mice in a total volume of 0.2 ml PBS. Five days later, samples of the SP, LN from the recipients were collected for cryosection. For immunofluorescent staining, slides were fixed in cold acetone for 10 minutes, and then washed for 10 minutes in PBS. Slides were stained with a PE-conjugated anti-mouse CD3, B220 or CD11c antibody (BioLegend). The sections were counter-stained with 1.0 µg/ml 4′, 6-Diamidino-2-phenylindole dihydrochloride (DAPI, Sigma) in PBS for 20 minutes at room temperature in the dark. Fluorescent cells on sections were visualized under Olympus CK2 fluorescence microscope.

### Murine GvHD model

Bone marrow cells (BMC) were obtained from BALB/c mice followed by red blood cell lysis. Splenic mononuleocytes (SPMNC) were isolated by Ficoll gradient centrifugation from Balb/C mice. In the GvHD group, 1×10^7^ BMC and 2×10^7^ SPMNC in a total volume of 0.2 ml PBS were injected into lethally irradiated (9Gy) C57BL/6 mice through lateral tail vein. MSCs/eGFP or MSCs/CCR7 (5×10^5^) were co-injected into GvHD mice, which were defined as GvHD+MSCs/eGFP and GvHD+MSCs/CCR7 groups of mice respectively.

The condition of the animals was monitored daily. The degrees of systemic acute GvHD were assigned a score from 0 to 2 for each of 5 GvHD parameters: weight loss, activity, fur-ruffling, kyphosis and skin lesions. Scores ranged from 0 (minimum) to 10 (maximum) [20]. Animals that developed clinical symptoms of GvHD (40% weight loss, hunched posture, fur loss, reduced mobility, tachypnea) were sacrificed in their homecage by CO_2_ inhalation to effect and an end point of survival was recorded for all GvHD mice. At different time points after infusion, mice were sacrificed by CO_2_ inhalation, and specimens from at least three recipient mice per group were collected for experiments.

### T cell proliferation assay

T cells in spleens of the three groups of mice were isolated with pan-T isolation kit (MiltenyiBiotec) according to the manufacturer’s instructions. T lymphocytes (5×10^5^/well) were planted in the 96-wells plate in RPMI 1640 supplemented with 20% FBS, and 100 U/mL IL-2 (Peprotech). The total volume was 200 µl per well. Plate was maintained at 37°C for 5 days and pulsed with 5 µCi of ^3^H-thymidine deoxyribonucleoside/ml for an additional 18 hours. Cells were then harvested onto glass fiber filters and radioactivity was measured on a Wallac Microbeta Trilux 1450-02P.

### Cytotoxicity assay

Cytotoxicity assay was performed with lactate dehydrogenase cytotoxicity detection kit (BioVision). T cells in spleens were isolated with pan-T isolation kit (MiltenyiBiotec). Cells were then incubated with 4×10^4^ EL4 leukemia cells for 12 hours on the 96-well plates. The wells of low control contain 4×10^4^ EL4 cells only in medium and the wells of high control contain 4×10^4^ EL4 cells in 1% Triton X-100. The absorbance at 450 nm, which represents the values of LDH release, was measured in an enzyme-linked immunosorbent assay (ELISA) reader. The cytotoxicity (%) = (Test Sample-low control)/(High control-low control)×100.

### Regulatory T cells (Tregs) detection

The Tregs (CD4^+^FoxP3^+^) populations in the spleens were detected by using Mouse Th17/Treg Phenotyping Kit (BD Pharmingen) according tothe manufacture instructions.

### Statistical analysis

The Kaplan-Meier product-limit method was used to calculate survival curve. Differences between groups in survival studies were determined using log-rank statistics. For all other data, a Student’s t test was used to analyze differences between groups, and results were considered significant if the *P* value was less than or equal to 0.05.

## Results

### CCR7 expression in MSCs

**Figure 1:**
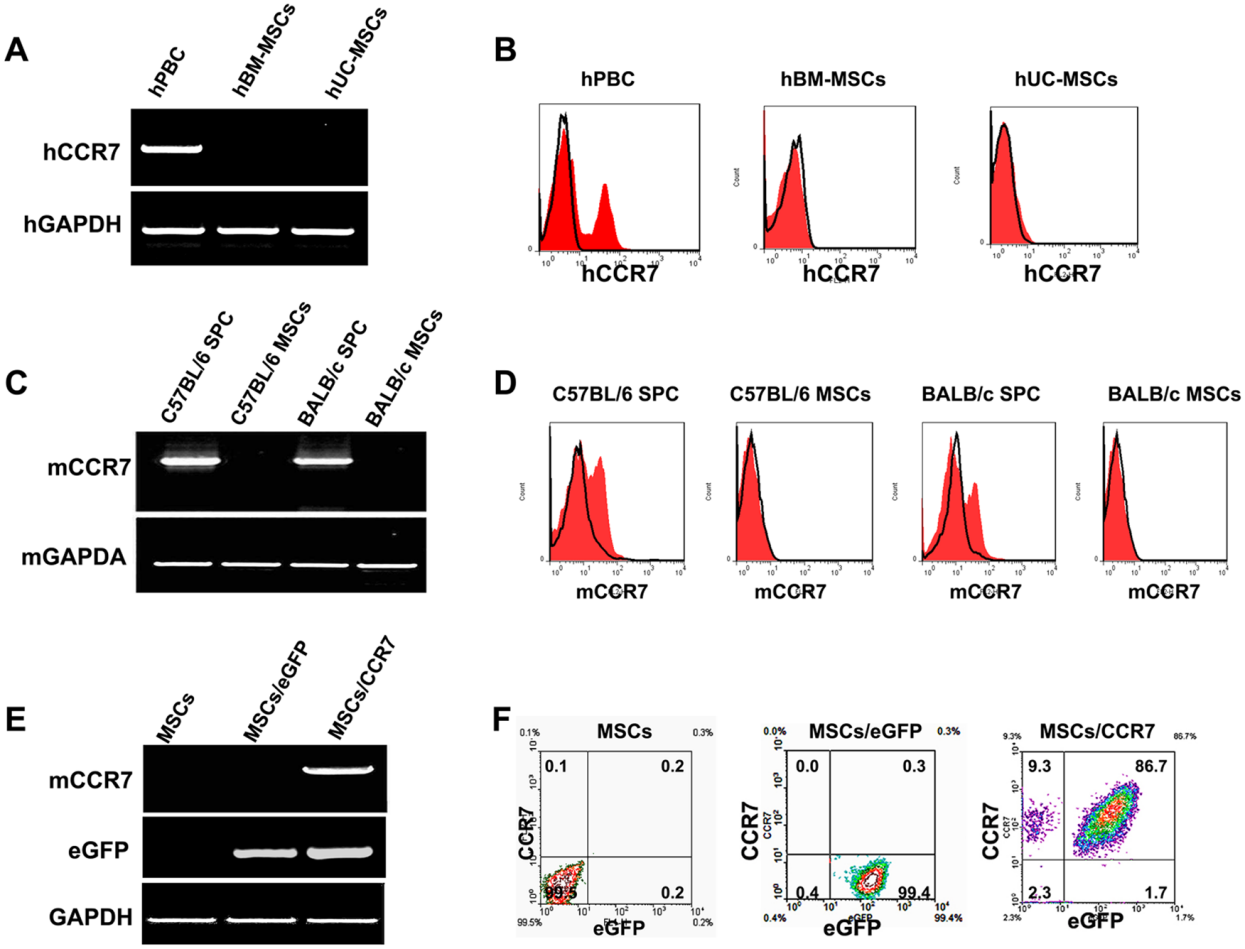
CCR7 expression in murine and human MSCs. (A) RT-PCR analysis of CCR7 mRNA expression level in the human primary MSCs derived from bone marrow (hBM-MSCs) and umbilical cord (hUC-MSCs), human peripheral blood cells (hPBC) served as positive control. Human housekeeping gene of GAPDH acted as internal standard control. (B) FCM analysis of CCR7 protein on the cell surface of human BM-MSCs and UC-MSCs. (C)RT-PCR analysis of CCR7 mRNA level in the primary MSCs, splenic mononucleocytes (SPC) served as positive controls. Murine housekeeping gene of GAPDH acted as internal standards control. (D)FCM analysis of CCR7 protein on the cell surface of SPC and murine MSCs derived from BALB/c and C57BL/6 mice. (E) Murine CCR7 gene was engineered into murine MSCs by lentiviral vector transduction. RT-PCR analysis of CCR7 and eGFP mRNA expression in the MSCs, MSCs/eGFP and MSCs/CCR7. (F) FCM analysis of CCR7 protein on the cell surface of the MSCs, MSCs/eGFP and MSCs/CCR7. Data are representative of three independent experiments.

CCR7 can guide various types of immune cells to SLOs [21]–[26]. Both hBM-MSCs and hUC-MSCs at passage 5 expressed traceable CCR7 mRNA detected by RT-PCR and FCM assay (Fig. 1A, 1B). Similarly, CCR7 was negligible in murine MSCs (Fig. 1C, 1D). Then, we transduced MSCs from C57BL/6 mouse strain with lentivirus carrying CCR7 gene. This group of cells was defined as MSCs/CCR7 in the following experiments. MSCs transduced with the control lentivirus were also referred as negative control (MSCs/eGFP). CCR7 can be successfully detected only in MSCs/CCR7at mRNA level detected by RT-PCR assay, and at cell surface protein level examined by FCM. The eGFP gene and encoded protein could be both detected in the MSCs/eGFP and MSCs/CCR7 (Fig. 1E, F).

### iNOS expression in MSCs/CCR7

**Figure 2:**
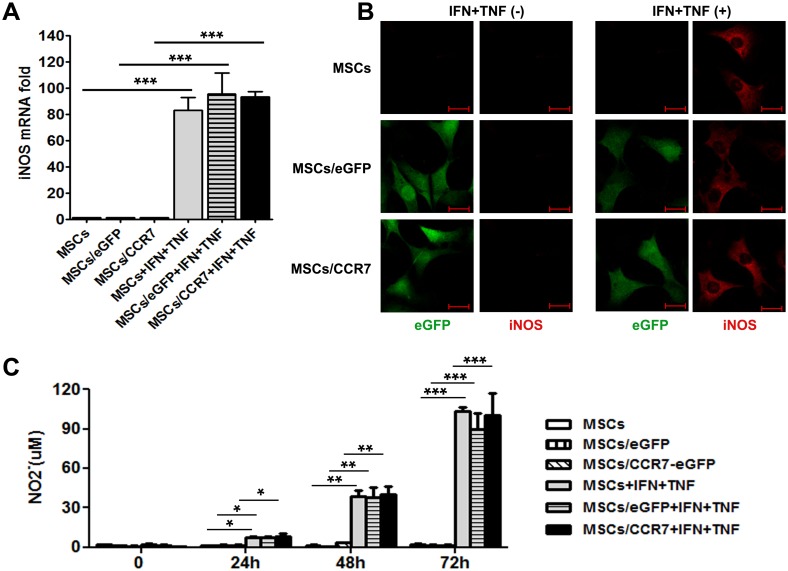
iNOS expression in MSCs/CCR7 is elicit by inflammatory cytokines. (A) iNOS expression was examined by real-time PCR in the MSCs, MSCs/eGFP and MSCs/CCR7 without or with IFNγ plus TNFα treatment. (B) iNOS expression was examined by in situ immunofluorescence staining. (C) MSCs, MSCs/eGFP and MSCs/CCR7 were stimulated with IFNγ plus TNFα. NO in the supernatant was examined by Griess assay. Data shown are mean±S.D. of a representative of 3 independent experiments. **P<0.01 and ***P<0.001 when compared with the cells without cytokine stimulation.

Previous investigations revealed that NO is solely generated by iNOS in murine MSCs and plays a critical role for their immunoregulatory function exerting [19], [27], [28]. We examined the iNOS level in MSCs, MSCs/eGFP and MSCs/CCR7. Contrasted to the cells without stimulation, iNOS mRNA increased nearly 100 folds in the three types of MSCs provoked by IFNγ plus TNFα for 72 hours (Fig. 2A). In situ immunofluorescence staining results confirmed that iNOS expression could be elicited by inflammatory cytokine stimulation (Fig. 2B). Though there were little dose without stimulation, the NO levels in the supernatant of three groups of MSCs were dramatically increased under inflammatory stimulation in a time dependent way (Fig. 2C).

### Immunomodulatory capacity of MSCs/CCR7 *in vitro*

**Figure 3:**
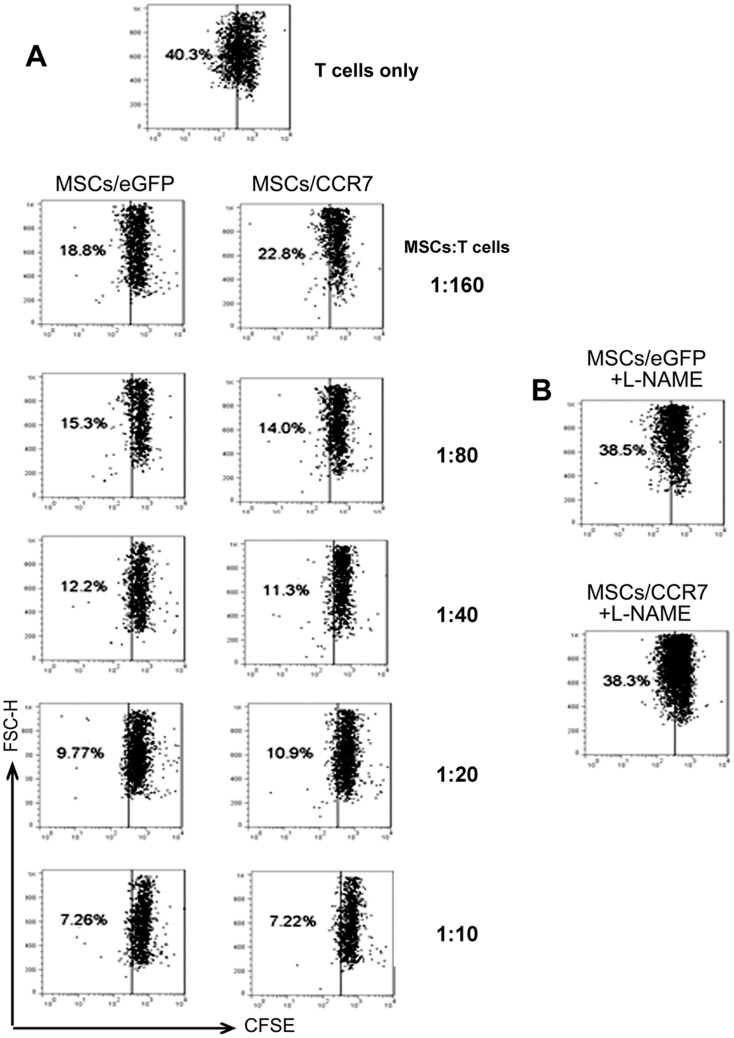
MSCs/CCR7 inhibit T cells proliferation in a dose dependant manner. (A) CFSE labeled CD3^+^T cells were cultured with MSCs at different ratios in the presence of PMA plus ION for 48 hours. T cell proliferation as indicated by the reduction in CFSE intensity was analyzed by FCM. (B) L-NMMA was added at the beginning of co-culture (MSCs:T cells = 1∶10). The cells were subjected to FCM for T-cell proliferation detection. Numbers adjacent indicate percentage of cells in the gate. Data are representative of three independent experiments.

Next, we examined whether CCR7 carrying MSCs still have immunomodulatory capacity *in vitro*. CFSE labeled CD3^+^T cells in the presence of PMA plus ION were cultured for 48 hours. The CFSE on the T cells were significantly diluted with proliferation. Co-culture of MSCs/eGFP or MSCs/CCR7 inhibited T cell proliferation in a dose dependant manner (Fig. 3A). NG-monomethyl-L- arginine acetate salt (L-NMMA, 2 mM),the iNOS inhibitor, was added to the T cell proliferation assay system (MSCs:T cells = 1∶10). As expected, L-NMMA treatment reinstated the T-cell proliferation in the co-culture assay (Fig. 3B). These results indicated that MSCs/CCR7 have similar immunomodulatory function in a NO-dependent manner as MSCs/eGFP.

### MSCs/CCR7 relocated at the appropriate T cell-rich zones within SLOs

**Figure 4:**
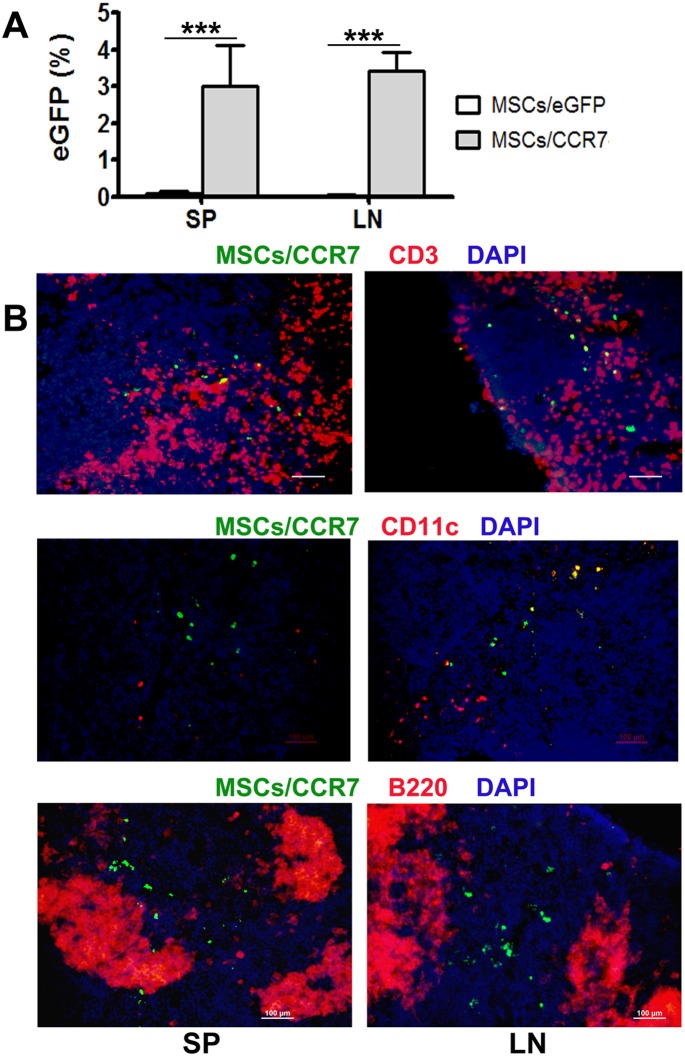
MSCs/CCR7 relocate within the T cell-rich zones of SLOs. (A)MSCs/eGFP or MSCs/CCR7 were intravenously injected into GvHD mice. Five days later, the proportion of eGFP^+^ cells in spleen (SP) and lymphoid nodes (LN) were examined by FCM. *, *p*<.005. (B) Cryosection slices of SP and LN of the recipients were immunofluorescent stained with CD3 antibody (red, upper panel), CD11c antibody (red, middle panel) or B220 antibody (red, lower panel), then counterstained with DAPI (blue). Bar = 100 µm. Data were representative of three independent experiments.

SLOs are constructed with T cells, B cells, antigen-presenting cells (APCs), stromal cells and vascular supplies. CCR7programmed MSCs migrate to and concentrate in SLOs after infusion (Fig. 4A). However, the precise anatomic sites in SLOs that the transfused MSCs/CCR7 found are crucial for their *in vivo* function exerting. Interestingly, our immunofluorescent staining results revealed that MSCs/CCR7 relocated at the right position of T cell-rich zones (relocated withCD3^+^T lymphocytes and CD11c^+^dentritic cells nearby, but far from B220^+^ B cell follicle, Fig. 4B–D). This set a foundation for MSCs/CCR7 exerting potent immunoregulatory activity on the T cell immune response.

### MSCs/CCR7 Co-infusion potently inhibited the lethality of GvHD

Donor naïve T cells initially traffic to the recipient SLOs when GvHD occurs, then they undergo activation, expansion, and subsequent migration to peripheral target organs [29], [30]. Since MSCs/CCR7 relocated at the right position of T cell-rich zones within SLOs, we next tested their *in vivo* activities in GvHD model, which is a representative T-cells response disease.

**Figure 5:**
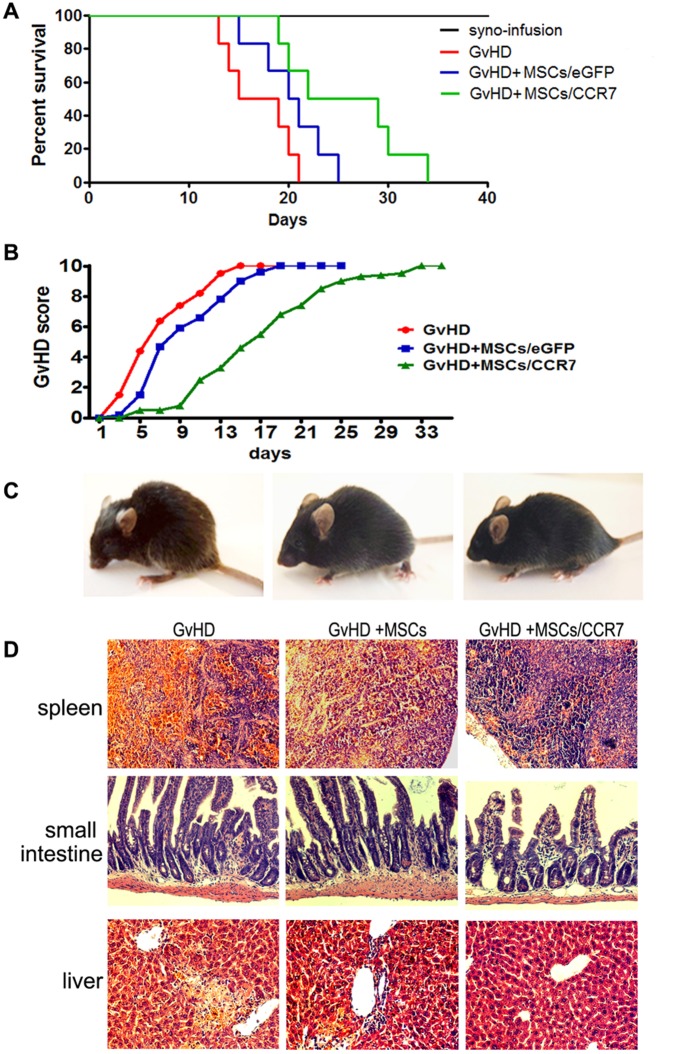
MSCs/CCR7 infusion remarkably inhibits the development of GvHD. (A) Survival curve of each group of mice (n = 12). (B) The degree of systemic GvHD scores of mice (n = 12). (C) The representative clinical experience of the three groups of mice.(D) The representative pathologic changes at day 14. Original magnification, ×200. Data were representative of three independent experiments.

Co-injection of 5×10^5^ MSCs did not prolong the survival time of GvHD mice. Captivatingly, co-injection of 5×10^5^ MSCs/CCR7 significantly enhanced the survival of the recipients (Fig. 5A). Recipient animals were evaluated using a validated clinical GvHD scoring system. Comparing with the mice ofGvHD and GvHD+MSCs groups, mice of GvHD+MSCs/CCR7 group also had considerable alleviated scores (Fig. 5B–C). These results demonstrated that MSCs/CCR7-eGFP were more efficient in immunomodulation, presumably due to the pivotal relocation site they hold in SLOs.

Pathologic examination confirmed that MSCs/CCR7 co-infusion declined the lymphocyte infiltration in the liver, small intestine. Comparing to the obvious lymphocyte existence in the spleens of the MSCs/CCR7 treated mice, there was large area of cellular atrophy and necrosis in the spleens of GvHD and GvHD+MSCs groups of mice (Fig. 5D).

### MSCs/CCR7 Co-infusion made the T cells in SLOs more naïve like and thus control of T cells trafficking from SLOs to the target organs

**Figure 6:**
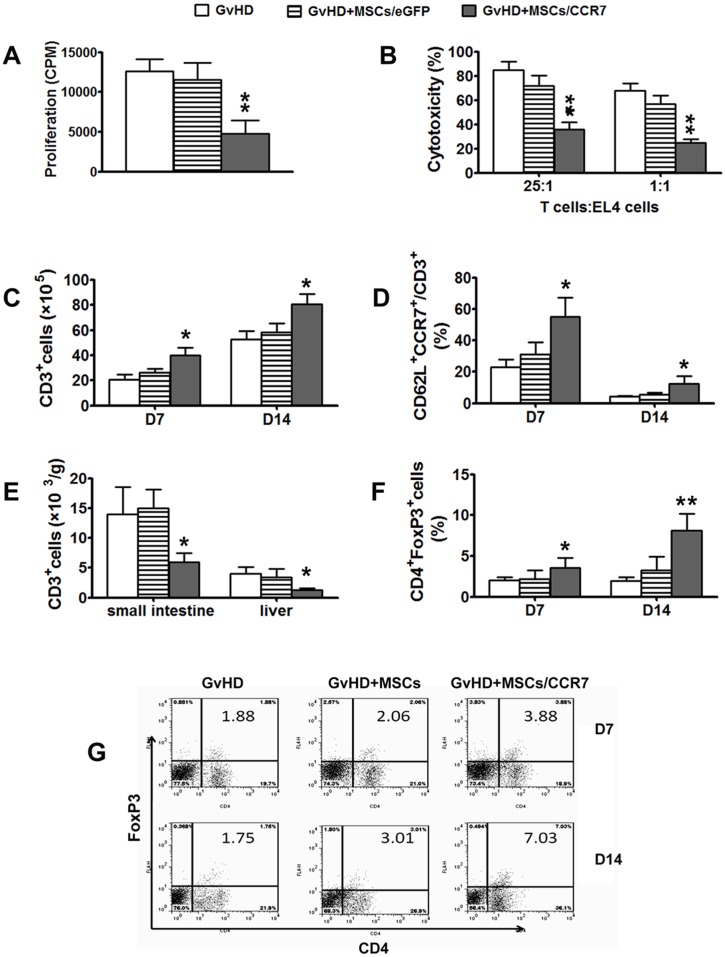
MSCs/CCR7 co-infusion makes the T cells more naïve like and thus coerce them stay in SLOs from trafficking to the target organs. (A) Splenic T cells were collected for proliferation capacity detection by ^3^H-thymidine uptake. (B) The cytotoxity of the splenic T cells of the three groups of mice. (C) The absolute numbers of the CD3^+^T cells in spleens of the three groups of mice, which were got by multiplying the nucleocyte numbers with the CD3^+^ cell percentage examined by FCM; (D) The proportion of the CD62L^+^CCR7^+^ cells in CD3^+^ T lymphocytes; (E) The absolute numbers of the CD3^+^ cells in the small intestines and livers of the three groups of mice. (F) The proportion of CD4^+^FoxP3^+^ Tregs in spleens of the three groups of mice; (G)The respective FCM results in (F) Data shown are mean±S.D. of a representative of 3 independent experiments.*P<0.05 when compared with the GvHD group of mice.

We further investigate the mechanism of *in vivo* immunoregulatory effects of MSCs/CCR7. Since MSCs/CCR7 relocated at the right position of T cell-rich zones within SLOs after infusion, as expected, the proliferations of T cells in SP were significantly restrained by MSCs/CCR7 co-infusion (Fig. 6A). Moreover, their cytotoxicities were also significantly suppressed (Fig. 6B).

CD3^+^T cells in SP were increased gradually as GvHD developing. Noteworthily, in contrast to the lower proliferation and lower cytotoxity of T cells, there were higher CD3^+^T cells in SP of the MSCs/CCR7 co-infusion group of mice (Fig. 6C). Further examination revealed that the increased T cells were more naïve like (the increased proportion of CD62L^+^CCR7^+^in CD3^+^cells) (Fig. 6D). Similar results were found in the LN, MLN and PP (data not shown). On the other hand, the CD3^+^ T cells infiltrated in the liver and small intestine declined (Fig. 6E). Tregs is a natural “suppressor” of the immune system. Our data showed that the proportion of Tregs remarkably increased in SP of the GvHD+MSCs/CCR7 group of mice (Fig. 6F).

## Discussion

As the clinical GvHD-therapy result of MSCs is not as satisfactory as expected in multicenter phase III clinical studies [31]–[34]. The aim of our present study is to further investigate the mechanism of enhanced *in vivo* immunomodulatory of CCR7 carrying MSCs.

CCR7 can guide various types of cell to SLOs, where generate immune responses or induce tolerance. As one kind of professional APCs, DCs are the most potent initiator of *in vivo* immune responses [35]–[37]. Upon maturation, DCs upregulate CCR7 expression and migrate from the peripheral tissues to the T-cell regions of SLOs, followed by instigating T-cell activation [21]–[25]. Regulatory T cells (Tregs) are instrumental to induce and maintain tolerance in transplantation immune response [38]–[40]. Moreover, Tregs mature in the SLOs of the recipients. Tregs induce allo-tolerance by interacting with APCs and T cells, which process requires their proper homing to the lymphoid tissues. CCR7 expression is important not only for Treg homing to the draining LN, but also for optimal Tregs suppressive function exerting [25].

Since murine MSCs and human MSCs scarcely express CCR7 at the mRNA level and cell surface protein level [41]–[45], we introduce CCR7 gene into murine MSCs by lentivirus infection. To our excitement, CCR7 carrying MSCs can target migrate to and relocate at the appropriate T cell-rich zones within SLOs, which set a foundation for MSCs/CCR7 to shape T cell immune response *in vivo*. Owing to the pivotal relocation sites, MSCs/CCR7 at the same dosage displayed enhanced effect than normal MSCs in prolonging the survival and alleviating the clinical scores of GvHD mice (Fig. 5A–C).

T lymphocytes are classified into four different subsets. Expressing CD62L and CCR7, Naïve T and central memory T cells can home to SLOs. Activated by APCs, effector memory T and effector T cells lose the expression of CD62L and CCR7, emigrate from SLOs into the peripheral inflammatory tissues [46]–[48]. Our results showed that MSCs/CCR7 infusion dramatically made T cells in SLOs less proliferous and cytotoxic. MSCs/CCR7 made donor T lymphocyte in SLOs more ‘naïve like’ (the higher expression levels of CD62L and CCR7 molecules on the CD3^+^T lymphocytes) (Fig. 6D). The increased naïve phenotype may give explanation to the increased donor T cells in SLOs and less lymphocytes infiltration in the peripheral target organs. Therefore, it was justifiable that MSCs/CCR7 made the T cells maintain in SLOs (Fig. 6C), and decrease infiltration in GvHD target organs (Fig. 6E). These results mean MSCs/CCR7 infusion spoiling the fourth supplemental Billingham’s tenets to inhibit GvHD development.

Consistent with the recent studies, the immunomodulatory effect of MSCs/CCR7 was not innate, and it could be induced by inflammatory stimulation (Fig. 2). This may confer the inducible modulatory activity of MSCs/CCR7 *in vivo*. In the other words, acting as potent inflammation holder, MSCs/CCR7 might calm down the high immune process quickly and keep quiet in the normal physiological circumstance as well.

Tregs is a natural “suppressor” of the immune system [49], [50]. It is generally recognized that Tregs treatment can reduce GvHD lethality [51], [52]. In our study, MSCs/CCR7 infusion significantly increased the Tregs population, which joined forces in making the T cells more naïve like in SLOs.

## Conclusion

Though CCR7-expressing MSCs exhibit equal modulatory activities as MSCs on T cell immune response in a NO-dependent manner *in vitro*, they can relocate at the appropriate T cell-rich zones within SLOs after infusion. MSCs/CCR7 co-infusion potently made the T cells in SLOs more naïve like, thus coerce T cells stay in SLOs from trafficking to the target organs. Through spoiling the fourth supplemental Billingham’s tenets, MSCs/CCR7 potently inhibited the development of GvHD. The study here provides a novel therapeutic strategy of MSCs/CCR7 infusion at a low dosage to give potent immunomodulation effect for clinical immune disease therapy.

## References

[1] Billingham RE (1966–1967) The biology of graft-versus-host reactions. Harvey Lect 62:21–78. 4875305

[2] Kim YM, Sachs T, Asavaroengchai W, Bronson R, Megan Sykes M, et al (2003) Graft-versus-host disease can be separated from graft-versus-lymphoma effects by control of lymphocyte trafficking with FTY720. J Clin Invest 111:659–669. 12618520 10.1172/JCI16950 PMC151899

[3] Taylor PA, Ehrhardt MJ, Lees CJ, Tolar J, Weigel BJ, et al (2007) Insights into the mechanism of FTY720 and compatibility with regulatory T cells for the inhibition ofgraft-versus-host disease (GVHD). Blood 110:3480–8. 10.1182/blood-2007-05-087940 PMC2200903 17606761

[4] Kataoka H, Ohtsuki M, Shimano K, Mochizuki S, Oshita K, et al (2005) Immunosuppressive activity of FTY720, sphingosine 1-phosphate receptor agonist: II. Effect of FTY720 and FTY720-phosphate on host-versus-graft and graft-versus-host reaction in mice. Transplant Proc 37:107–109. 15808562 10.1016/j.transproceed.2004.12.287

[5] Sackstein R (2006) A revision of Billingham’s tenets: The central role of lymphocyte migration in acute graft-versus-host disease. Biol Blood Marrow Transplant 12:2–8. 16399577 10.1016/j.bbmt.2005.09.015

[6] Coghill JM, Carlson MJ, Panoskaltsis-Mortari A, West ML, Burgents JE, et al (2010) Separation of graft-versus-host disease from graft-versus-leukemia responses by targeting CC-chemokine receptor 7 on donor T cells. Blood 115:4914–22. 20185583 10.1182/blood-2009-08-239848 PMC2890182

[7] Teschner D, Distler E, Wehler D, Frey M, Marandiuc D, et al (2014) Depletion of naive T cells using clinical grade magnetic CD45RA beads: a new approach for GVHD prophylaxis. Bone Marrow Transplant 49:138–144. 23933765 10.1038/bmt.2013.114

[8] Mafi R, Hindocha S, Mafi P, Griffin M, Khan WS (2011) Sources of adult mesenchymal stem cells applicable for musculoskeletal applications - a systematic review of the literature. Open Orthop J 5:242–8. 21886689 10.2174/1874325001105010242 PMC3149887

[9] Paunescu V, Deak E, Herman D, Siska IR, Tanasie G, et al (2007) In vitro differentiation of human mesenchymal stem cells to epithelial lineage. J Cell Mol Med 11:502–8. 17635641 10.1111/j.1582-4934.2007.00041.x PMC3922356

[10] Oswald J, Boxberger S, Jørgensen B, Feldmann S, Ehninger G, et al (2004) Mesenchymal stem cells can be differentiated into endothelial cells in vitro. Stem Cells 22:377–84. 15153614 10.1634/stemcells.22-3-377

[11] Tran TC, Kimura K, Nagano M, Yamashita T, Ohneda K, et al (2011) Identification of human placenta-derived mesenchymal stem cells involved in re-endothelialization. J Cell Physiol 226:224–35. 20658518 10.1002/jcp.22329

[12] Jiang XX, Zhang Y, Liu B, Zhang SX, Wu Y, et al (2005) Human mesenchymal stem cells inhibit differentiation and function of monocyte-derived dendritic cells. Blood 105:4120–4126. 15692068 10.1182/blood-2004-02-0586

[13] Németh K, Leelahavanichkul A, Yuen PS, Mayer B, Parmelee A, et al (2009) Bone marrow stromal cells attenuate sepsis via prostaglandin E(2)-dependent reprogramming of host macrophages to increase their interleukin-10 production. Nat Med 15:42–49. 19098906 10.1038/nm.1905 PMC2706487

[14] Prockop DJ, Oh JY (2012) Medical Therapies with Adult stem/Progenitor Cells (MSCs): A Backward Journey From Dramatic Results In vivo to the Cellular and Molecular Explanations. Journal of Cellular Biochemistry 113:1460–1469. 22213121 10.1002/jcb.24046 PMC4147853

[15] Papatriantafyllou M (2011) Tracking immune cells on the lymph node map. Nat Rev Immunol 11:644. 10.1038/nri3068 21904386

[16] Ruddle NH, Akirav EM (2009) Secondary Lymphoid Organs: Responding to Genetic and Environmental Cues in Ontogeny and the Immune Response. J Immunol-183:2205–2212. 10.4049/jimmunol.0804324 PMC2766168 19661265

[17] Li H, Jiang Y, Jiang X, Guo X, Ning H, et al (2014) CCR7 guides migration of mesenchymal stem cell to secondary lymphoid organs: A novel approach to separate GvHD from GvL effect. Stem Cells 32:1890–1903. 24496849 10.1002/stem.1656

[18] Guo Z, Li H, Li X, Yu X, Wang H, et al (2006) In vitro characteristics and in vivo immunosuppressive activity of compact bone-derived murine mesenchymal progenitor cells. Stem Cells 24:992–1000. 16644925 10.1634/stemcells.2005-0224

[19] Sato K, Ozaki K, Oh I, Meguro A, Hatanaka K, et al (2007) Nitric oxide plays a critical role in suppression of T-cell proliferation by mesenchymal stem cells. Blood 109:228–34. 10.1182/blood-2006-02-002246 16985180

[20] van Den Brink MR, Moore E, Horndasch KJ, Crawford JM, Hoffman J, et al (2000) Fas-deficient lpr mice are more susceptible to graft-versus-host disease. J Immunol 164:469–480. 10.4049/jimmunol.164.1.469 10605044

[21] Johnson LA, Jackson DG (2014) Control of dendritic cell trafficking in lymphatics by chemokines. Angiogenesis 17:335–345. 24232855 10.1007/s10456-013-9407-0

[22] von Andrian UH, Mempel TR (2003) Homing and cellular traffic in lymph nodes. Nat Rev Immunol 3:867–878. 14668803 10.1038/nri1222

[23] Chauhan SK, Saban DR, Dohlman TH, Dana R (2014) CCL-21 Conditioned Regulatory T Cells Induce Allotolerance through Enhanced Homing to Lymphoid Tissue. J Immunol 192:817–823. 24337379 10.4049/jimmunol.1203469 PMC3947335

[24] Viola A, Contento LV, Molon B (2006) T cells and their partners: The chemokine dating agency. Trends Immunol 27:421–427. 16860609 10.1016/j.it.2006.07.004

[25] Schneider MA, Meingassner JG, Lipp M, Moore HD, Rot A (2007) CCR7 is required for the in vivo function of CD4+ CD25+ regulatory T cells. J Exp Med 204:735–745. 17371928 10.1084/jem.20061405 PMC2118557

[26] Förster R, Schubel A, Breitfeld D, Kremmer E, Renner-Müller, et al. (1999) CCR7 coordinates the primary immune response by establishing functional microenvironments in secondary lymphoid organs. Cell. 99:23–33. 10.1016/s0092-8674(00)80059-8 10520991

[27] Su J, Chen X, Huang Y, Li W, Li J, et al (2014) Phylogenetic distinction of iNOS and IDO function in mesenchymal stem cell-mediated immunosuppression in mammalian species. Cell Death Differ 21:388–396. 24162664 10.1038/cdd.2013.149 PMC3921585

[28] Li W, Ren G, Huang Y, Su J, Han Y, et al (2012) Mesenchymal stem cells: a double-edged sword in regulating immune responses. Cell Death Differ 19:1505–1513. 22421969 10.1038/cdd.2012.26 PMC3422473

[29] Panoskaltsis-Mortari A, Price A, Hermanson JR, Taras E, Lees C, et al (2004) In vivo imaging of graft-versus-host-disease in mice. Blood 103:3590–3598. 14715632 10.1182/blood-2003-08-2827

[30] Beilhack A, Schulz S, Baker J, Beilhack GF, Wieland CB, et al (2005) In vivo analyses of early events in acute graft-versus-host disease reveal sequential infiltration of T-cell subsets. Blood106:1113–1122. 10.1182/blood-2005-02-0509 PMC1895168 15855275

[31] Lin Y, Hogan WJ (2011) Clinical Application of MesenchymalStemCells in the Treatment and Prevention of Graft-versus-Host Disease. AdvHematol 427863. 10.1155/2011/427863 PMC3235491 22190941

[32] Martin I, Baldomero H, Tyndall A, Niederwieser D, Gratwohl A (2010) A survey on cell and engineered tissue therapies in Europe in 2008. Tissue Eng Part A 16:2419–2427. 20184422 10.1089/ten.TEA.2010.0056

[33] Tyndall A. (2011) Successes and failures of stem cell transplantation in autoimmune diseases. Hematology Am SocHematolEduc Program 2011:280–284. 10.1182/asheducation-2011.1.280 22160046

[34] Tyndall A, Houssiau FA (2010) Mesenchymal stem cells in the treatment of autoimmune diseases. Ann Rheum Dis 69:1413–1414. 20650875 10.1136/ard.2010.132639

[35] Miller MJ, Wei SH, Parker I, Cahalan MD (2002) Two-photon imaging of lymphocyte motility and antigen response in intact lymph node. Science 296:1869–1873. 12016203 10.1126/science.1070051

[36] Stoll S, Delon J, Brotz TM, Germain RN (2002) Dynamic imaging of T cell-dendritic cell interactions in lymph nodes. Science 296:1873–1876. 12052961 10.1126/science.1071065

[37] Mempel TR, Henrickson SE, Von Andrian UH (2004) T-cell priming by dendritic cells in lymph nodes occurs in three distinct phases. Nature 427:154–159. 14712275 10.1038/nature02238

[38] Verneris MR (2013) Natural killer cells and regulatory T cells: how to manipulate a graft for optimal GVL. Hematology Am SocHematolEduc Program 2013:335–341. 10.1182/asheducation-2013.1.335 PMC4020013 24319201

[39] Wolf D, Hochegger K, Wolf AM, Rumpold HF, Gastl G, et al. (2005) CD4+CD25+ regulatory T cells inhibit experimental anti-glomerular basement membrane glomerulonephritis in mice. J Am SocNephrol. 2005. 10.1681/ASN.2004100837 15788479

[40] Parmar S, Liu X, Tung SS, Robinson SN, Rodriguez G, et al (2014) Third-party umbilical cord blood-derived regulatory T cells prevent xenogenic graft-versus-host disease. Cytotherapy 16:90–100. 24480547 10.1016/j.jcyt.2013.07.009 PMC4124936

[41] Brooke G, Tong H, Levesque JP, Atkinson K (2008) Molecular trafficking mechanisms of ultipotentmesenchymal stem cells derived from human bone marrow and placenta. Stem Cells. Dev 17:929–940. 10.1089/scd.2007.0156 18564033

[42] Sordi V, Malosio ML, Marchesi F, Mercalli A, Melzi R, et al (2005) Bone marrow mesenchymal stem cells express a restricted set of functionally active chemokine receptors capable of promoting migration to pancreatic islets. Blood 106:419–427. 15784733 10.1182/blood-2004-09-3507

[43] Honczarenko M, Le Y, Swierkowski M, Ghiran I, Glodek AM, et al (2006) Human bone marrow stromal cells express a distinct set of biologically functional chemokine receptors. Stem Cells 24:1030–1041. 16253981 10.1634/stemcells.2005-0319

[44] Chamberlain G, Wright K, Rot A, Ashton B, Middleton J (2008) Murine Mesenchymal Stem Cells Exhibit a Restricted Repertoire of Functional Chemokine Receptors: Comparison with Human. PLoS ONE3:e2934. 10.1371/journal.pone.0002934 PMC2488395 18698345

[45] Blazar BR, Taylor PA, McElmurry R, Tian L, Panoskaltsis-Mortari A, et al (1998) Engraftment of severe combined immune deficient mice receiving allogeneic bone marrow via in utero or postnatal transfer. Blood 92:3949–3959. 9808589

[46] Ho LP, Yit PS, Ng LH, Linn YC, Zhao Y, et al (2013)The road to memory: an early rest for the long journey. J Immunol 191:5603–5614. 24184558 10.4049/jimmunol.1301175

[47] Unsoeld H, Pircher H (2005) Complex memory T-cell phenotypes revealed by coexpression of CD62L and CCR7. J Virol 79(7):4510–4513. 15767451 10.1128/JVI.79.7.4510-4513.2005 PMC1061544

[48] Worbs T, Förster R (2007) A key role for CCR7 in establishing central and peripheral tolerance. Trends Immunol 28:274–80. 17462953 10.1016/j.it.2007.04.002

[49] Beres AJ, Drobyski WR (2013) The role of regulatory T cells in the biology of graft versus host disease. Front Immunol 24:163. 10.3389/fimmu.2013.00163 PMC3690651 23805140

[50] Haase D, Starke M, Puan KJ, Lai TS, Rotzschke O (2012) Immune modulation of inflammatory conditions: regulatory T cells for treatment of GvHD. Immunol Res 53:200–212. 22418725 10.1007/s12026-012-8267-9

[51] Karakhanova S, Munder M, Schneider M, Bonyhadi M, Ho AD, et al (2006) Highly efficient expansion of human CD4+CD25+ regulatory T cells for cellular immunotherapy in patients with graft-versus-host disease. J Immunother 29:336–349. 16699377 10.1097/01.cji.0000203080.43235.9e

[52] Duramad O, Laysang A, Li J, Ishii Y, Namikawa R (2011) Pharmacologic expansion of donor-derived, naturally occurring CD4(+)Foxp3(+) regulatory T cells reduces acute graft-versus-host disease lethality without abrogating the graft-versus-leukemia effect in murine models. Biol Blood Marrow Transplant 17:1154–1168. 21145405 10.1016/j.bbmt.2010.11.022

